# A new approach to prevent radiation-induced xerostomia using intraglandular injection of mitochondria-boosting agents

**DOI:** 10.1186/s12885-024-12582-2

**Published:** 2024-07-11

**Authors:** Mohammad Farhadi, Pedram Fadavi, Saleh Mohebbi, Farzad Taghizadeh-Hesary

**Affiliations:** 1 ENT and Head and Neck Research Center and Department, The Five Senses Health Institute, School of Medicine, Iran University of Medical Sciences, Tehran, Iran; 2 Department of Radiation Oncology, Iran University of Medical Sciences, Tehran, Iran; 3 Skull Base Research Center, The Five Senses Health Institute, School of Medicine, Iran University of Medical Sciences, Tehran, Iran; 4 Breast Health Cancer Research Center, School of Medicine, Iran University of Medical Sciences, Tehran, Iran

**Keywords:** Drug delivery, Mitochondria, Radiotherapy, Xerostomia

## Abstract

Radiotherapy in patients with head and neck cancer fairly leads to xerostomia, profoundly affecting their quality of life. With limited effective preventive and therapeutic methods, attention has turned to exploring alternatives. This article outlines how intraglandular injection of mitochondria-boosting agents can serve as a potential strategy to reduce salivary acinar damage. This method can contribute to the thoughtful development of study protocols or medications to reduce radiation-induced salivary glands damage.

## Main text

Radiotherapy (RT) is the mainstay of treatment in patients with head and neck cancer [1]. Dry mouth (xerostomia) is one of the most common complaints observed in patients following head and neck RT [2]. Studies reveal that even with modern RT techniques, such as IMRT, a significant number of patients may be impacted by dry mouth [3]. Radiation-induced xerostomia (RiX) can occur early during the course of RT, with approximately a 50–60% decline in salivary flow within the first week of RT [4]. This adverse effect stems from direct acinar damage followed by collagen deposition in the salivary glands, transforming the glandular tissue into a fibrotic state and reducing the efficacy of therapeutic interventions [5]. Recovery from dry mouth typically requires at least six months [6], and it has been reported that over 64% of patients continue to complain of moderate to severe xerostomia even three years post-treatment [7]. A recent study demonstrated that only 18% of treated patients experienced complete recovery of xerostomia after a median follow-up of 37 months [8]. This condition significantly disrupts speech, swallowing, and oral hygiene, thus profoundly affecting the patient’s quality of life [9]. Therefore, efficient methods to prevent RiX are of crucial importance.

This issue has urged researchers worldwide to evaluate different strategies to reduce the incidence or severity of RiX [10]. There is still no confident method to prevent dry mouth. Surgical interventions have been applied to displace the submandibular glands to the submental space or forearm away from the radiation fields [11, 12]; however, this procedure might be associated with surgical complications and does not preserve the parotid glands from radiation. The sole FDA-approved xerostomia preventive method, amifostine, faces limited acceptance due to serious side effects [13]. In addition, some studies raised concern that amifostine may not be effective in the prevention of xerostomia in patients who receive concomitant chemotherapy [14]. Regarding the treatment of RiX, the available approaches use saliva substitutes and cholinergic agents (pilocarpine and cevimeline), which are limited in broad-spectrum clinical application due to short-term and severe side effects, respectively [15]. As such, the quest for effective preventive methods regarding RiX remains crucial.

RiX primarily arises from the generating free radicals and acinar cell damage [16]. Given this concept, several studies have tried to evaluate the efficacy of different sorts of antioxidants to prevent RiX. While antioxidants, such as α-lipoic acid (ALA), are highly reactive to free radicals and can prevent tissue damage and dysfunction [16], their effectiveness in preventing RiX may be limited. In addition, concerns about the tumor-protective effects of antioxidants would further restrict their clinical application during the RT course [17]. Due to these limitations, alternative agents are required to diminish the radiation effects on protecting salivary glands.

Emerging research has indicated that bolstering normal tissue mitochondria can mitigate RT side effects [18]. For example, Mohamed and Said demonstrated that coenzyme Q10 (CoQ10) (a mitochondria-boosting agent) could prevent the radiation-induced bowel toxicity in a rat model [19]. In a separate animal study, low-dose CoQ10 could provide protection against radiation-induced ovarian damage [20]. These benefits might be due to the following mechanistic pathways: (1) As noted in our previous study, activated mitochondria can reduce the radiation effects by scavenging the released reactive oxygen species (ROSs) [21]; (2) Besides, the mitochondrial enhancement would restore the adenosine-triphosphate (ATP) content of salivary cells, supporting the function DNA-preserving proteins (e.g., PARP-1, XRCC1, ATM, and DNA ligases), which are reliant on ATP molecules [22]; (3) Cell proliferation is an energy-consuming process [23]. Hence, mitochondrial activation can improve the normal tissue capacity to divide and replace the damaged tissues. The first mechanism reflects the “radioprotective effect” of mitochondrial-boosting agents, while the second and third mechanisms reflect their “radiomitigating effect” [24].

Therefore, boosting the salivary glands’ acinar cells’ mitochondrial metabolism can potentially reduce the risk of acinar damage, thereby xerostomia, following radiation. In the following, two questions need to be answered: (Q1) Which agents can be applied to boost the mitochondrial function of salivary glands acinar cells? (Q2) What is the best method to deliver the selected mitochondria-activating agent to the salivary glands? Systemic or local?

R1) Mitochondrial metabolism relies on several cofactors; for example, CoQ10, α-lipoic acid, and L-carnitine. These cofactors have been applied in the supplements and medicines to improve the mitochondrial function [25]. Among these, CoQ10 is of special interest, and is more commonly applied in clinical settings. Idebenone is a synthetic, water-soluble form of CoQ10 applied to treat hereditary mitochondrial disorders [26]. In addition to the three mechanisms mentioned above, CoQ10 can further protect the acinar cells from radiation effects by its intrinsic antioxidant effects [27]. Other potential agents to improve mitochondrial biogenesis are resveratrol [28], mitochondria-targeted antioxidants (e.g., Mito-Tempo, mitoquinone [MitoQ], and 10-(6′-plastoquinonyl) decyltriphenylphosphonium [SKQ1]) [29], mitochondrial biogenesis modulators (e.g., PGC-1α activators). Selecting a single agent simplifies monitoring of its impact on mitochondrial biogenesis, ensuring focused and effective intervention. Among the available choices, idebenone is likely the optimal choice. FDA-approved for various human diseases, its superior tolerability, water solubility, and potent antioxidant properties make the idebenone a compelling option over CoQ10 [26]. In addition, the activation of idebenone in the mitochondrial membrane, cytosol, and mitochondrial matrix underscores its advantages over CoQ10 [30].

**Fig. 1:**
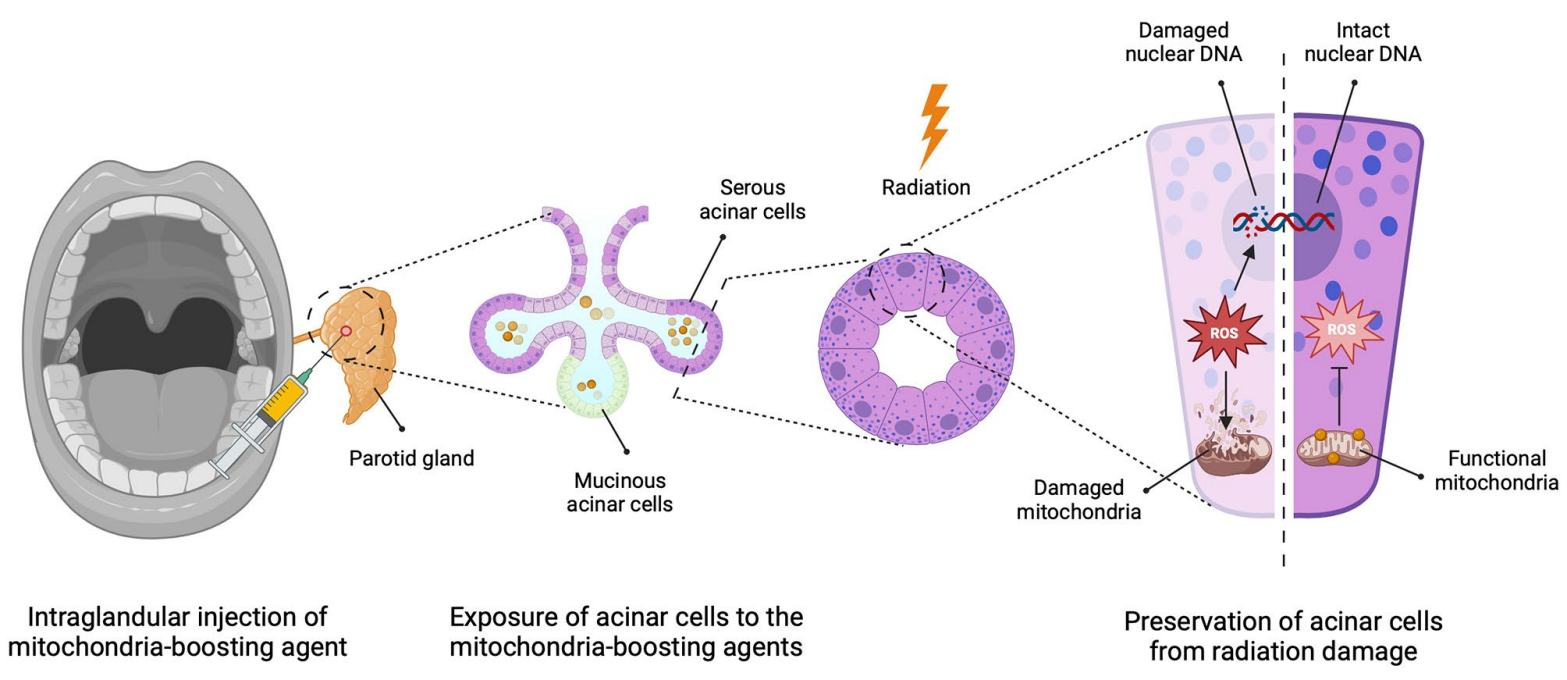
Prevention of radiation-induced xerostomia using intraglandular injection of mitochondria-boosting agents

R2) Systemic administration of mitochondria activators might interfere with the radiation effects on the cancer cells by directly activating the cancer cells’ mitochondria [21]. In addition, cancer cells can improve their mitochondrial content by hijacking the functional mitochondria from the nearby normal cells through the intercellular nanoscale tubes [31]. With this background in mind, we propose a new technique to prevent the radiation damage to the salivary glands (Fig. 1):

In this approach, the at-risk major salivary glands are identified based on RT dosimetric planning and salivary gland tolerance. The mitochondria-enhancing agent is injected into the at-risk salivary glands before primary RT and regularly during radiation. Intraglandular injections entail administering medication directly into the salivary glands. This approach offers the benefit of direct access, minimizing systemic side effects common with numerous therapies. Additionally, it is a time-efficient approach that necessitates lower dosages for injection. For patients undergoing RT, the intraglandular injection approach is preferred over the intraductal approach as it avoids the need to administer medication through the inflamed mucosa. This method bypasses potential disruptions to the procedure or challenges with tolerability. Applying a non-surgical transcutaneous approach can assist in reducing the morbidity of the procedures [32]. The benefit of an ultrasound guide can assist in better exploring the salivary glands [32]. Regular application of anti-inflammatory ointments or creams during radiation therapy can facilitate the use of transcutaneous injections throughout RT. Eventually, this method would enhance the acinar cells’ tolerability to the released ROS and improve their capability to repair the radiation-induced DNA damage (discussed above). This approach has the potential to inform the deliberate design of study protocols to mitigate radiation-induced salivary gland damage. To formulate comprehensive protocols for human studies, conducting animal-based studies is crucial to address key knowledge gaps, including determining the optimal agent, dosage, and administration sequence.

## Conclusions

The impact of RiX on patients with head and neck cancer underscores the critical need for effective preventive and therapeutic approaches. Current preventive interventions, including surgical repositioning of glands and medicines, fall short due to associated complications and limited efficacy. Similarly, the use of antioxidants in preventing RiX presents challenges in balancing radioprotective effects and tumor protection concerns. As a promising alternative, enhancing salivary gland cells’ mitochondria offers multifaceted benefits. Activated mitochondria scavenge reactive oxygen species, restore ATP levels, and bolster cell proliferation, collectively mitigating radiation effects on salivary glands. Further exploration of mitochondrial-boosting agents is crucial in addressing the challenges posed by RiX and enhancing patients’ quality of life.

## Data Availability

No datasets were generated or analysed during the current study.

## Abbreviations

ALA: α-lipoic acid
ATM: Ataxia telangiectasia mutated
ATP: Adenosine triphosphate
CoQ10: Coenzyme Q10
IMRT: Intensity-modulated radiation therapy
MitoQ: Mitoquinone
PAPR1: Poly (ADP-ribose) polymerase 1
PGC-1α: Peroxisome proliferator-activated receptor-gamma coactivator 1 alpha
RiX: Radiation-induced xerostomia
ROS: Reactive oxygen species
RT: Radiotherapy
SKQ1: 10-(6′-plastoquinonyl) decyltriphenylphosphonium
XRCC1: X-ray repair cross complementing 1

## References

[1] Ameri A, Norouzi S, Sourati A, Azghandi S, Novin K, Taghizadeh-Hesary F. Randomized trial on acute toxicities of weekly vs three‐weekly cisplatin‐based chemoradiation in head and neck cancer. Cancer Rep. Jan. 2022;5(1):e1425. 10.1002/cnr2.1425. 10.1002/cnr2.1425 PMC8789619 34101389

[2] Berger T, Noble DJ, Shelley LEA, McMullan T, Bates A, Thomas S (2022). Predicting radiotherapy-induced xerostomia in head and neck cancer patients using day-to-day kinetics of radiomics features. Phys Imaging Radiat Oncol.

[3] Snider JW (2020). Sticky stuff: xerostomia in patients undergoing head and neck radiotherapy-prevalence, prevention, and palliative care. Ann Palliat Med Vol.

[4] Pinna R, Campus G, Cumbo E, Mura I, Milia E (2015). Xerostomia induced by radiotherapy: an overview of the physiopathology, clinical evidence, and management of the oral damage. Ther Clin Risk Manag.

[5] Nam K et al. Jan., Copper chelation reduces early collagen deposition and preserves saliva secretion in irradiated salivary glands, *Heliyon*, vol. 10, no. 2, p. e24368, 2024, 10.1016/j.heliyon.2024.e24368. 10.1016/j.heliyon.2024.e24368 PMC10828693 38298614

[6] Jaguar GC, Prado JD, Campanhã D, Alves FA. Clinical features and preventive therapies of radiation-induced xerostomia in head and neck cancer patient: a literature review. Appl Cancer Res, 37, 1, 2017.

[7] Wijers OB, Levendag PC, Braaksma MM, Boonzaaijer M, Visch LL, Schmitz PI (2002). Patients with head and neck cancer cured by radiation therapy: a survey of the dry mouth syndrome in long-term survivors. Head Neck.

[8] Rades D, Warwas B, Gerull K, Pries R, Leichtle A, Bruchhage KL (2022). Prognostic factors for complete recovery from Xerostomia after Radiotherapy of Head-and-Neck cancers. Vivo.

[9] Nascimento ML, Farias AB, Carvalho AT, Albuquerque RF, Ribeiro LN, Leao JC. Impact of xerostomia on the quality of life of patients submitted to head and neck radiotherapy. Med Oral Patol Oral Cir Bucal, 24, 6, 2019. 10.4317/medoral.23131 PMC6901149 31655838

[10] Grundmann O, Fillinger JL, Victory KR, Burd R, Limesand KH. Restoration of radiation therapy-induced salivary gland dysfunction in mice by post therapy IGF-1 administration. BMC Cancer, 10, 1, 2010. 10.1186/1471-2407-10-417 PMC3087323 20698985

[11] Rieger J, Seikaly H, Jha N, Harris J, Williams D, Liu R (2005). Submandibular Gland Transfer for Prevention of Xerostomia after Radiation Therapy: swallowing outcomes. Arch Otolaryngol Neck Surg.

[12] Hagen R, Scheich M, Kleinsasser N, Burghartz M (2016). Two-stage autotransplantation of human submandibular gland: a novel approach to treat postradiogenic xerostomia. Eur Arch Otorhinolaryngol.

[13] Rades D, Fehlauer F, Bajrovic A, Mahlmann B, Richter E, Alberti W (2004). Serious adverse effects of amifostine during radiotherapy in head and neck cancer patients. Radiother Oncol.

[14] Gu J, Zhu S, Li X, Wu H, Li Y, Hua F. Effect of amifostine in head and neck cancer patients treated with radiotherapy: a systematic review and meta-analysis based on randomized controlled trials. PLoS ONE, 9, 5, 2014. 10.1371/journal.pone.0095968 PMC4008569 24788761

[15] Li Y, Li X, Pang R, Yang G, Tian M, Zhao T. Diagnosis, Prevention, and Treatment of Radiotherapy-Induced Xerostomia, *Rev. J Oncol*, vol. 2022, no. 7802334, 2022. 10.1155/2022/7802334 PMC9440825 36065305

[16] Liu Z, Dong L, Zheng Z, Liu S, Gong S, Meng L. Mechanism, Prevention, and treatment of Radiation-Induced Salivary Gland Injury related to oxidative stress. Antioxid Basel, 10, 11, 2021. 10.3390/antiox10111666 PMC8614677 34829539

[17] D’Andrea GM (2005). Use of antioxidants during chemotherapy and radiotherapy should be avoided. CA Cancer J Clin.

[18] Behnam B, Taghizadeh-Hesary F. Mitochondrial metabolism: a New Dimension of Personalized Oncology. Cancers, 15, 16, 2023. 10.3390/cancers15164058 PMC10452105 37627086

[19] Mohamed HA, Said RS. Coenzyme Q10 attenuates inflammation and fibrosis implicated in radiation enteropathy through suppression of NF-kB/TGF-β/MMP-9 pathways. Int Immunopharmacol. Mar. 2021;92:107347. 10.1016/j.intimp.2020.107347. 10.1016/j.intimp.2020.107347 33418245

[20] Tekin YB et al. May., Evaluation of the protective effect of coenzyme Q10 against x-ray irradiation‐induced ovarian injury, *J. Obstet. Gynaecol. Res*, p. jog.15966, 2024, 10.1111/jog.15966. 10.1111/jog.15966 38757238

[21] Taghizadeh-Hesary F, Houshyari M, Farhadi M (2023). Mitochondrial metabolism: a predictive biomarker of radiotherapy efficacy and toxicity. J Cancer Res Clin Oncol.

[22] Taghizadeh-Hesary F. Reinforcement by Tumor Microenvironment: the seventh ‘R’ of Radiobiology. Int J Radiat Oncol Biol Phys, 2023. 10.1016/j.ijrobp.2023.09.027 38032584

[23] VG AA, ME E, MC C (2012). Mitochondrial regulation of cell cycle and proliferation. Antioxid Redox Signal.

[24] Montoro A, Obrador E, Mistry D, Forte GI, Bravatà V, Minafra L (2023). Radioprotectors, Radiomitigators, and Radiosensitizers.

[25] Pagano G, Aiello Talamanca A, Castello G, Cordero MD, d’Ischia M, Gadaleta MN (2014). Current experience in testing mitochondrial nutrients in disorders featuring oxidative stress and mitochondrial dysfunction: rational design of chemoprevention trials. Int J Mol Sci.

[26] Suárez-Rivero JM, Pastor-Maldonado CJ, Povea-Cabello S, Álvarez-Córdoba M, Villalón-García I, Munuera-Cabeza M. Coenzyme Q10 analogues: benefits and challenges for therapeutics. Antioxidants, 10, 2, 2021. 10.3390/antiox10020236 PMC7913973 33557229

[27] Silva SVE, Gallia MC, Luz J, Rezende AA, Bongiovanni GA, Araujo-Silva G. Antioxidant effect of Coenzyme Q10 in the Prevention of oxidative stress in Arsenic-treated CHO-K1 cells and possible participation of Zinc as a Pro-oxidant Agent. Nutrients, 14, 16, 2022. 10.3390/nu14163265 PMC9412518 36014770

[28] Jardim FR, Rossi FT, Nascimento MX, Silva Barros RG, Borges PA, Prescilio IC (2018). Resveratrol and Brain Mitochondria: a review. Resveratrol Brain Mitochondria Rev Mol Neurobiol.

[29] Sacks B, Onal H, Martorana R, Sehgal A, Harvey A, Wastella C. Mitochondrial targeted antioxidants, mitoquinone and SKQ1, not vitamin C, mitigate doxorubicin-induced damage in H9c2 myoblast: pretreatment vs. co-treatment. BMC Pharmacol Toxicol, 22, 1, 2021. 10.1186/s40360-021-00518-6 PMC8447656 34530934

[30] Gueven N, Woolley K, Smith J. Border between natural product and drug: comparison of the related benzoquinones idebenone and coenzyme Q10. Redox Biol. Apr. 2015;4:289–95. 10.1016/j.redox.2015.01.009. 10.1016/j.redox.2015.01.009 PMC4803797 25625583

[31] Saha T, et al. Intercellular nanotubes mediate mitochondrial trafficking between cancer and immune cells. Nat Nanotechnol. Jan. 2022;17(1):98–106. 10.1038/s41565-021-01000-4. 10.1038/s41565-021-01000-4 PMC10071558 34795441

[32] Almansoori AA, Hariharan A, Cao UMN, Upadhyay A, Tran SD. Drug Therapeutics Delivery to the Salivary Glands: Intraglandular and Intraductal Injections, in *Cell Biology and Translational Medicine, Volume 20*, vol. 1436, K. Turksen, Ed., in Advances in Experimental Medicine and Biology, vol. 1436., Cham: Springer Nature Switzerland, 2023, pp. 119–130. 10.1007/5584_2023_765. 10.1007/5584_2023_765 36809639

